# Circadian Oscillation of the Lettuce Transcriptome under Constant Light and Light–Dark Conditions

**DOI:** 10.3389/fpls.2016.01114

**Published:** 2016-07-27

**Authors:** Takanobu Higashi, Koh Aoki, Atsushi J. Nagano, Mie N. Honjo, Hirokazu Fukuda

**Affiliations:** ^1^Graduate School of Life and Environmental Sciences, Osaka Prefecture UniversitySakai, Japan; ^2^Faculty of Agriculture, Ryukoku UniversityOtsu, Japan; ^3^Core Research for Evolutional Science and Technology – Japan Science and Technology AgencyKawaguchi, Japan; ^4^Center for Ecological Research, Kyoto UniversityOtsu, Japan; ^5^Graduate School of Engineering, Osaka Prefecture UniversitySakai, Japan; ^6^Precursory Research for Embryonic Science and Technology, Japan Science and Technology AgencyKawaguchi, Japan

**Keywords:** circadian clocks, enrichment, lettuce, oscillations, plant factories, transcriptome

## Abstract

Although, the circadian clock is a universal biological system in plants and it orchestrates important role of plant production such as photosynthesis, floral induction and growth, there are few such studies on cultivated species. Lettuce is one major cultivated species for both open culture and plant factories and there is little information concerning its circadian clock system. In addition, most of the relevant genes have not been identified. In this study, we detected circadian oscillation in the lettuce transcriptome using time-course RNA sequencing (RNA-Seq) data. Constant light (LL) and light–dark (LD) conditions were used to detect circadian oscillation because the circadian clock has some basic properties: one is self-sustaining oscillation under constant light and another is entrainment to environmental cycles such as light and temperature. In the results, 215 contigs were detected as common oscillating contigs under both LL and LD conditions. The 215 common oscillating contigs included clock gene-like contigs *CCA1* (*CIRCADIAN CLOCK ASSOCIATED 1*)-like, *TOC1* (*TIMING OF CAB EXPRESSION 1*)-like and *LHY* (*LATE ELONGATED HYPOCOTYL*)-like, and their expression patterns were similar to those of *Arabidopsis*. Functional enrichment analysis by GO (gene ontology) Slim and GO Fat showed that the GO terms of response to light stimulus, response to stress, photosynthesis and circadian rhythms were enriched in the 215 common oscillating contigs and these terms were actually regulated by circadian clocks in plants. The 215 common oscillating contigs can be used to evaluate whether the gene expression pattern related to photosynthesis and optical response performs normally in lettuce.

## Introduction

The circadian clock is a universal biological system in plants that has been well-studied in *Arabidopsis* (Harmer et al., 2000; Haydon et al., 2011; Farré and Weise, 2012). The circadian clock consists of three components: input, central oscillator and output pathways. Each component involves a number of genes. *PHY*s (*PHYTOCHROME*s), *CRY*s (*CRYPTOCHROME*s), and *PHOT*s (*PHOTOTROPIN*s) are the best-known light receptor genes of input pathways and transmit external light stimuli to the central oscillator (Christie, 2007; Pruneda-Paz and Kay, 2010). *CCA1* (*CIRCADIAN CLOCK ASSOCIATED 1*), *LHY* (*LATE ELONGA TED HYPOCOTYL*), *TOC1* (*TIMING OF CAB EXPRESSION 1*) and *PRR*s (*PSEUDO-RESPONSE REGULATORs*) are central oscillator genes – they are known as clock genes and are important in generating the circadian rhythm (Gardner et al., 2006; Nakamichi et al., 2010, 2012). *CO* (*CONSTANS*) and *FT* (*FLOWERING LOCUS T*) are downstream genes of circadian clocks in the output pathways and regulate flowering (Suárez-López et al., 2001; Corbesier et al., 2007). Recent study indicated that circadian clocks control several physiological events such as photosynthesis and stress response (Dodd et al., 2005, 2014; Covington et al., 2008; Lai et al., 2012). In addition, controlling circadian clocks has potential to enhance productivity of cultivated species such as broccoli, petunia and lettuce by effects on growth, floral induction and pest resistance (Goodspeed et al., 2013; Fenske et al., 2015; Higashi et al., 2015; Tanigaki et al., 2015; Thiruvengadam et al., 2015; Zhai et al., 2015). These results show that controlling circadian clocks has potential to achieve high efficiency in plant production. Because circadian clocks are readily controlled by external stimuli, especially light and temperature, one target among plant production systems is closed-type plant factories as environmental parameters are readily tightly controlled (Rensing and Ruoff, 2002; Fukuda et al., 2013).

Lettuce is a typical crop in closed-type plant factories because it is suitable for hydroponic culture and can be cultivated under a low-light conditions (Li et al., 2016; Moriyuki and Fukuda, 2016; Wang et al., 2016). Lettuce is a diploid (2*n* = 18) species with genome size of 2.7 Gb (Truco et al., 2007, 2013); however, many genes including clock genes have not have been identified. Therefore, there are few studies on the circadian clock in lettuce. One application suited to analyzing the behavior of circadian clocks without genome information is RNA sequencing (RNA-Seq), a revolutionary tool for omics studies (Wang et al., 2009). RNA-Seq can obtain transcriptome information and oscillating genes (or contigs) can be detected from time-course RNA-Seq data (Nagano et al., 2012; Matsuzaki et al., 2015; Schick et al., 2016). To detect the oscillating contigs generated by the circadian clock, time-course transcriptome data of lettuce cultivated under both constant light (LL) and 12 h light and 12 h dark (LD) conditions are needed. Circadian clocks have some basic properties: one is self-sustaining oscillation under constant light or dark conditions and the other is entrainment to environmental fluctuations such as light and temperature (Nakamichi et al., 2004). Thus, oscillating contigs generated by circadian clocks cannot correctly be detected using only one of the two light conditions.

In this study, we tried to detect the oscillating contigs generated by circadian clocks using time-course transcriptome data of lettuce, which is a typical crop in closed-type plant factories. We performed the experiments under LL and LD conditions and detected the oscillating contigs common to both. In addition, we also used homology and gene ontology (GO) analysis to estimate the function of the oscillating contigs in lettuce.

## Materials and Methods

### Plant Materials and Growing Systems

Lettuce plants (*Lactuca sativa* L. cv. Frill Ice from Snow Brand Seed, Co. Ltd, Hokkaido, Japan) were grown in a closed cultivation system. Seeds were sown on a water-laden urethane sponge in a tray (400 mm × 280 mm × 70 mm) filled with water and incubated for a week under fluorescent light [photosynthetic photon flux density (PPFD) = 250–450 μmol m^-2^ s^-1^]. The environmental parameters of germination and growing conditions were 22°C and 12-h light and 12-h darkness (12L:12D).

After 1 week, seedlings were transplanted to the multistage hydroponic system. The light sources used were red, green and blue LEDs (660, 520, and 450 nm, respectively; Shibasaki, Inc., Saitama, Japan). Cultivation was performed using a Deep Flow Technique hydroponic system. A submersible pump was placed in a tank containing the culture medium to maintain constant circulation at 10–15 L min^-1^, and a total of three cultivation beds (2720 mm × 640 mm × 150 mm; Sanki Keiso, Co. Ltd, Saitama, Japan) were filled with the culture medium at a specified constant pH and electric conductivity (EC). In each bed, three cultivation panels (885 mm × 590 mm × 30 mm; M Hydroponic Research, Co. Ltd, Aichi, Japan) were installed with open planting holes and root zones at a water depth of 90 mm. The inter-hole distance was 70 mm across the length and 100 mm across the width. The cultivation medium was composed of tap water and fertilizer (N:P_2_O_5_:K_2_O:CaO:MgO = 10:8:27:0:4 and 11:0:0:23:0; Otsuka House No. 1 and 2, respectively; Otsuka Chemical, Co. Ltd, Osaka, Japan) at pH 6.0 and EC 2.0. The pH and EC settings were performed with reference to the Otsuka Chemical standard solution formulations. Transplanted seedlings were grown in the multistage hydroponic system for 15 days. The environmental parameters were 22°C, 50% relative humidity, 1000 μmol mol^-1^ CO_2_ concentration and LL or LD, with R:G:B = 120:40:40; total PPFD = 180–220 μmol m^-2^ s^-1^. In the LL experiment, light condition was set 12L:12D and switched to LL at 12 days after transplanting because the oscillating component disappears if cultivated continuously under LL condition for a long time (Nakamichi et al., 2004; Higashi et al., 2014).

We sampled the largest leaves every 2 h for 2 days, starting at 13 days and ending 15 days after transplanting. These leaves were immediately frozen in liquid nitrogen and stored at -80°C.

### RNA-Seq Assay and Data Analysis

We applied DNase treatment to reduce DNA contamination and isolated total RNA using an RNeasy Plant Mini Kit (Qiagen). Its quality was checked using an Agilent 2100 Bioanalyzer (Agilent Technologies, Palo Alto, CA, USA). RNA quantity control was performed using a Qubit^®^ 2.0 Fluorometer (Life Technologies, Carlsbad, CA, USA). We prepared a RNA-Seq library (Wang et al., 2011; Nagano et al., 2015). Then we obtained the sequence read files using a HiSeq 2000 sequencer (single end, 50 bp; Illumina, San Diego, CA, USA). These sequence data are available in the DDBJ Sequenced Read Archive^1^ under the accession numbers DRA004542 and DRA004561.

All reads of each sample were quality-checked by FastQC and mapped using RSEM (RNA-Seq by Expectation Maximization; Li and Dewey, 2011) with Bowtie2 software (Langmead and Salzberg, 2012) to the predicted transcriptome models in the NCBI database^2^. We confirmed about 90% of the bases were aligned to these models (Supplementary Table S1). By these processes, we obtained the data of expression levels of contig for each sample. Finally, we used the reads per kilobase per million mapped reads measure to normalize the expression for total read length and the number of sequencing reads.

Clock gene expression data of *Arabidopsis thaliana* were obtained from the Diurnal database^3^. We obtained time-course clock gene expression data at 4-h intervals using LLHC [LL combined with temperature of hot and cool (HC) at 12-h intervals] and LDHC (LD combined with HC) conditions. We normalized each expression level using their averages and standard deviations. The normalized expression level was calculated as the value of expression level minus average expression level, divided by the standard deviation.

### Detection of Oscillating Contigs

We utilized the molecular timetable method (Ueda et al., 2004; Higashi et al., 2016) to detect oscillating contigs for LL and LD conditions. This method can detect some genes with high amplitude and periodicity from the time-course transcriptome data. Furthermore, it can detect collective behavior of oscillating genes by developing expression profiles.

First we selected contigs whose expression indicated high amplitude and periodicity. The amplitude value (*a*) was calculated as the standard deviation divided by the average of expression level. To analyze periodicity, we prepared 1440 test cosine curves. These curves had different peaks (0–24 h) measured at 1-min increments. We fitted test cosine curves to data from each time-course transcriptome generated via RNA-Seq and calculated the correlation value (*r*) to identify the best-fitting cosine curve. The peak time of the best-fitting curve was estimated as the peak time for each contig. This estimated peak time was defined as the molecular peak time. We set the cut-off values of *a* = 0.15 and *r* = 0.8, according to values from Ueda et al. (2004). Then we normalized expression level of oscillating contigs using their averages and standard deviations. The normalized expression level was calculated as the value of expression level minus average expression level, divided by the standard deviation.

Second, we selected common contigs detected as oscillating contigs by the molecular timetable method under both LL and LD conditions. Hereby, we detected oscillating contigs independent of light conditions.

Finally, the oscillating contigs were submitted to the BLAST program to undertake GO analysis and improve reliability as the subsistent genes. The cut-off values were minimal E-value of 10^-20^ and minimal query cover and identity of 60% for BLASTn and BLASTx between oscillating contigs and the NCBI database. Oscillating contigs were initially submitted to BLASTn and then non-hit contigs were submitted to BLASTx.

### Calculation of Measurement Noise

We calculated the measurement noise for the expression profiles of common oscillating contigs, using a previous molecular timetable method (Ueda et al., 2004; Higashi et al., 2016). We calculated the measurement noise from the standard deviation of the difference between a real and an estimated expression of all selected oscillating contigs.

### Functional Categorization by GO Slim and GO Fat

Functional categorization by GO Slim was performed using the TAIR10 database^4^. Corresponding *Arabidopsis* orthologs for detected oscillating contigs were determined by KEGG database^5^ using several plant gene identifications obtained by BLAST result.

Functional categorization by GO Fat was performed using the DAVID database^6^. The GO Fat database contains more specific terms than the GO database (Dennis et al., 2003). We used *Arabidopsis* TAIR ID as input information for the oscillating contigs and used GO Fat with default status.

### Functional Enrichment Analysis

Functional enrichment analysis for oscillating contigs was performed using the Biological Networks Gene Ontology (BiNGO) software (Maere et al., 2005). BiNGO is an open-source Java tool and can be used as a Cytoscape plugin^7^ (Shannon et al., 2003). We also used *Arabidopsis* TAIR ID as the input information for the oscillating contigs and performed functional enrichment analysis with default status.

## Results

### Detection of Oscillating Contigs under Both LL and LD Conditions

**FIGURE 1:**
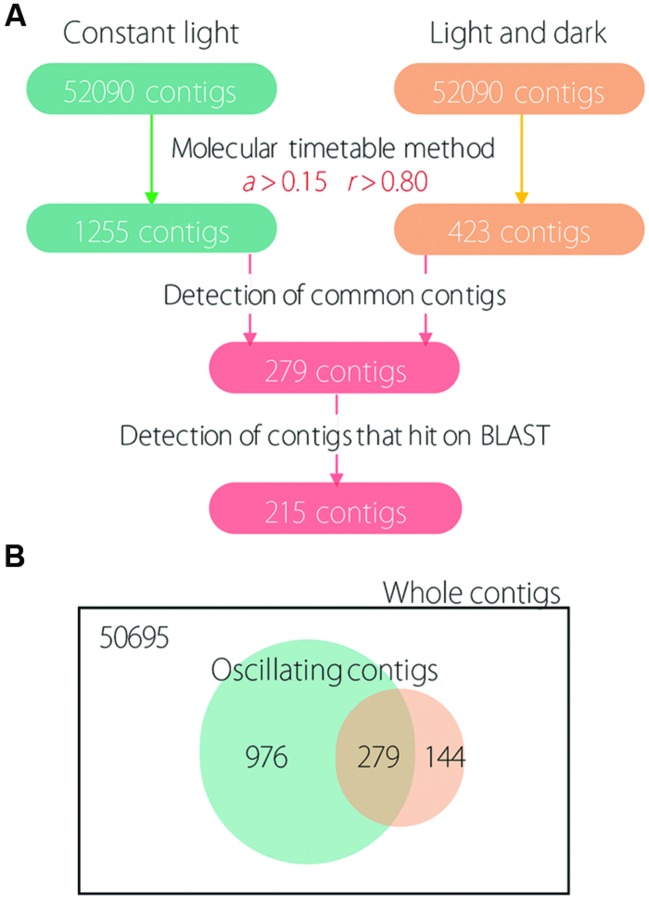
**Scheme of oscillating analysis and the number of detected contigs. (A)** Numbers in colored boxes indicate the detected number of contigs. **(B)** Venn diagram showing unique and shared contigs under LL and LD conditions.

First, we determined oscillating contigs for LL and LD conditions using the molecular timetable method. Under LL conditions, 1255 contigs were detected as oscillating (**Figure 1A**); under LD conditions, only 423 contigs were detected as oscillating. Next, we determined 279 common oscillating contigs for both LL and LD conditions; we used the BLAST program for functional analysis and to confirm identity of the subsistent genes. This resulted in 215 common oscillating contigs under both LL and LD conditions (Supplementary Table S2). As a result, 972 and 144 contigs were detected as unique contigs that oscillated only under LL or LD conditions, respectively; and 279 contigs were detected as shared contigs that oscillated under both LL and LD conditions (**Figure 1B**).

**FIGURE 2:**
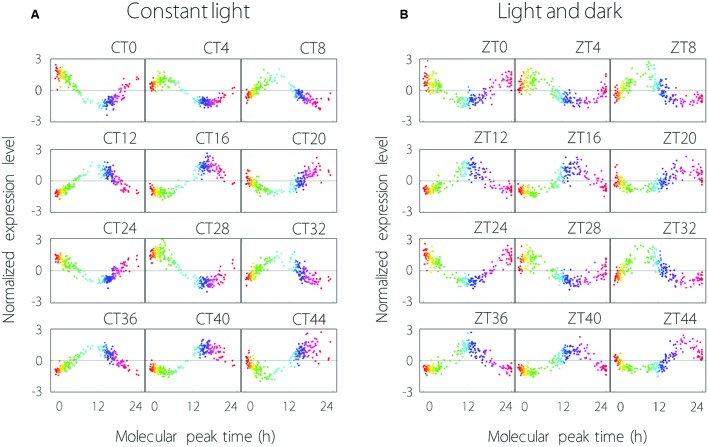
**Global analysis of 215 oscillating contigs using the molecular timetable method under LL (A) and LD (B) conditions. (A)** The time at the top right of the plot area indicates sampling time. CT0 (Circadian Time 0) is the time at the first sampling point. The range from blue to pink represents subjective night-time. Each plot indicates individual contig expression. **(B)** ZT0 (Zeitgeber Time 0) is the time at the first sampling point. The range from blue to pink represents night-time. Each plot indicates individual contig expression.

The expression profiles of the 215 common oscillating contigs for each time point are shown in **Figure 2**. We confirmed that the collective behavior of the 215 contigs showed stable periodicity, hence each common oscillating contig was expressed periodically under both LL and LD conditions. Measurement noise of expression profiles under LL conditions was in the range of 30–61% [mean ± standard deviation of 40 ± 6%], and under LD conditions was 39–57% (49 ± 5%). Thus, measurement noise tended to be larger under LD than LL conditions.

### Comparison of Expression Pattern between Lettuce Clock Gene-Like Contigs and *Arabidopsis* Clock Genes

**FIGURE 3:**
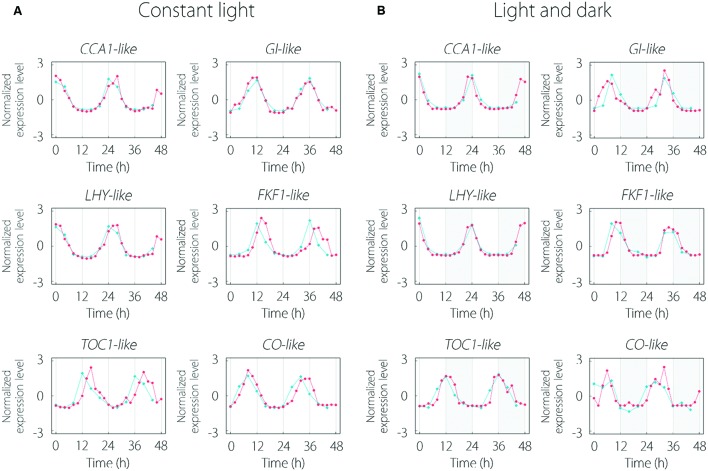
**Expression pattern of clock gene-like contigs under LL (A) and LD (B) conditions.** The red and blue lines indicate the expression patterns in lettuce and *Arabidopsis*, respectively. Clock gene expression data of *Arabidopsis thaliana* were obtained from the Diurnal database. Gray area in **(B)** represents the dark period.

Some clock gene-like contigs existed in 215 common oscillating contigs. *CCA1*, *LHY*, and *TOC1* are central oscillator genes in the circadian clock. *GI* (*GIGANTEA*) and *FKF1* (*FLAVIN-BINDING, KELCH REPEAT, F-BOX 1*) regulate flowering through regulation of transcription of *CO* gene. *CO* encodes the transcriptional regulator of *FT* and triggers floral induction. Every clock gene-like contig indicated periodic expression and these expression patterns were similar to those of *Arabidopsis* for LL and LD conditions (**Figure 3**).

### Functional Categorization and Enrichment Analysis

**FIGURE 4:**
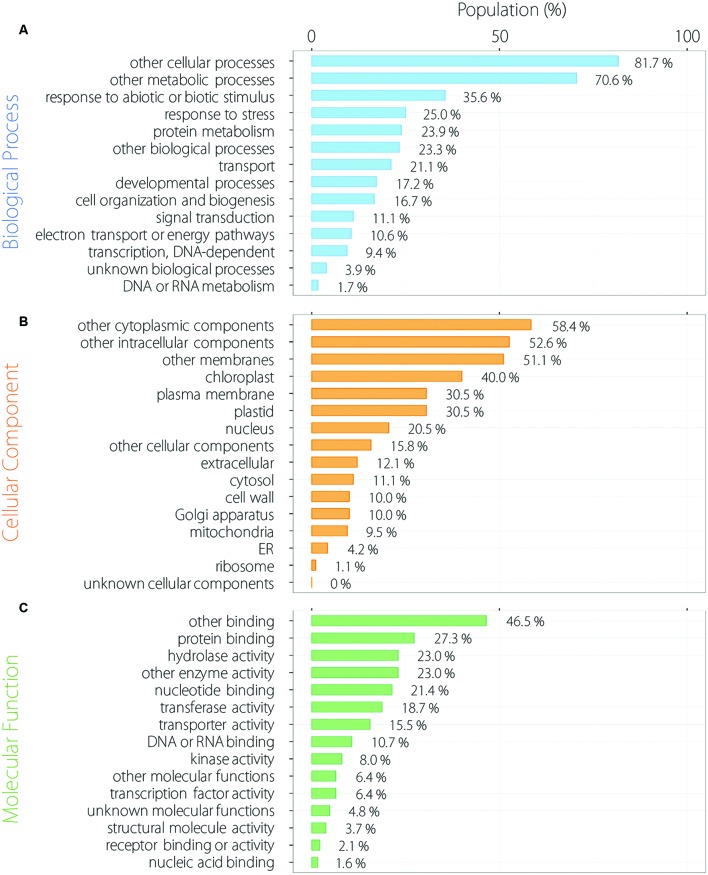
**Functional categorization of 215 oscillating contigs.** Biological process **(A)**, cellular component **(B)** and molecular function **(C)**.

We analyzed functional categorization of 215 common oscillating contigs using *Arabidopsis* orthologs by GO Slim. In the biological process (BP), 215 common oscillating contigs were especially enriched for the term of response to abiotic or biotic stimulus and response to stress except for other cellular and metabolic processes (**Figure 4A**). Contigs related to response to external stimulus accounted for a substantial fraction of the 215 common oscillating contigs. In the cellular component (CC), 215 common oscillating contigs were especially enriched for the terms of chloroplast and plastid related to photosynthesis (**Figure 4B**). In the molecular component (MC), 215 common oscillating contigs were especially enriched for terms of protein binding and hydrolase activity (**Figure 4C**). Furthermore, we analyzed more specific functional categorization using GO Fat. In each category, 215 common oscillating contigs were enriched for the terms related to light stimuli and photosynthesis, such as response to light stimulus, photosynthesis/light reaction, chloroplast part, photosystem, chlorophyll binding, and photoreceptor activity (Supplementary Table S3).

**FIGURE 5:**
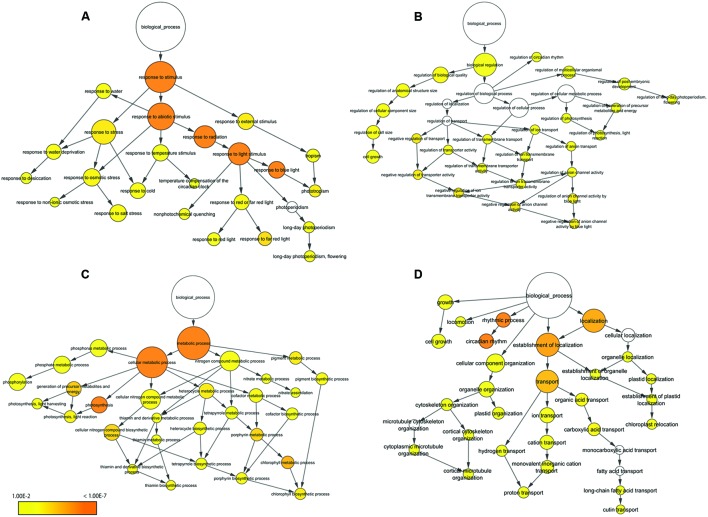
**Functional enrichment analysis of 215 oscillating contigs.** Response to stimulus **(A)**, biological regulation **(B)**, metabolic process **(C)** and other function **(D)**. The size of the circles represents the gene numbers. The color of each circle represents the enrichment *P*-value. The positions of nodes were changed manually.

We performed BP-specific enrichment analysis using BiNGO software to visualize the result of BP concerning which category was linked to application for plant production. In the terms related to response to stimulus, contigs were enriched describing response to stress, abiotic stimulus and external stimulus (**Figure 5A**). In particular, response to blue light was significantly enriched compared to red or far red light. In the terms related to biological regulation, contigs describing regulation of cell size, circadian rhythm, flowering and anion channel activity in response to blue light were enriched (**Figure 5B**); overall, this category showed low enrichment compared to other categories. In the terms related to metabolic process, contigs were enriched describing photosynthesis and chlorophyll metabolic process (**Figure 5C**). In addition, thiamin biosynthesis process – a major nutrient for lettuce and related to stress response – was also enriched. Of other functions, contigs were enriched describing circadian rhythm, transport and chloroplast and plastid localization (**Figure 5D**). In particular, rhythmic process and circadian rhythm were significantly enriched.

## Discussion

The molecular timetable method clearly showed fewer detected oscillating contigs under LD than LL conditions (**Figure 1**). This indicated that the expression pattern of contigs under LD conditions approximated cosine curves (as fitted using the molecular timetable method) in time-course expression data to detect periodicity. This is likely due to the ‘square wave’ in the application of the light cycle, in which plants receive an instantaneous change in light stimulus at the time of light-on and light-off. These instantaneous light stimuli can be considered to affect gene expression as some noise. In fact, *CCA1* expression was not stable for the LD cycle with the square wave; however, it was stable under the LD cycle with a cosine curve in lettuce (Higashi et al., 2014). The effect of the square wave was also evident in expression profiles of 215 common oscillating contigs. Although, expression profiles indicated periodicity for each light condition, measurement noise tended to be larger under LD than LL conditions. This suggests that the light cycle with a square wave caused some noise for gene expression in plants. The light cycle with a square wave may negatively affect plants because it is not present under natural conditions. In contrast, previous studies indicated that periodic gene expression was stable under fluctuating field conditions (Nagano et al., 2012; Matsuzaki et al., 2015; Higashi et al., 2016). In the present study, 144 contigs oscillated under LD but not under LL conditions (**Figure 1B**). This is not surprising, given that the periodicity of gene expression depends on light conditions and there was a clear difference between LL and LD conditions. In *Arabidopsis*, some CCA1 target genes indicated different expression patterns between LL and LD conditions (Nagel et al., 2015). In cyanobacteria, some genes whose expression exhibits periodicity under LL and LD conditions were detected by time-course transcriptional analysis (Toepel et al., 2008). The present study revealed that some periodic genes showed a differential expression pattern between LL and LD conditions. Furthermore, from the aspect of omics analysis, it was not always true that protein abundance indicated periodicity even though gene expression showed periodicity under LD condition in cyanobacteria (Waldbauer et al., 2012); however, they used only a light–dark specific analysis and did not consider a circadian clock system. In fact, coordination of transcriptome and metabolome by circadian clock system was revealed in mice and *Arabidopsis* (Fukushima et al., 2009; Eckel-Mahan et al., 2012). These omics studies suggest that the 215 common oscillating contigs detected in the lettuce transcriptome were probably coordinated by a circadian clock because they showed the self-sustaining oscillation characteristic of circadian clocks (Nakamichi et al., 2004; Higashi et al., 2014) and presumably the metabolome was coordinated on a periodic basis.

Clock gene-like contigs of lettuce existed in 215 common oscillating contigs and were expressed similarly to *Arabidopsis* under both LL and LD conditions. Although the expression period and peak phase had slight differences between lettuce and *Arabidopsis* under LL conditions, they seemed to depend on light quality. In a previous study, the period of circadian rhythm was altered by qualities such as red and blue light (Higashi et al., 2014). The differences in light quality might be shown in expression patterns. Furthermore, the expression pattern under LD conditions was noisy and did not clearly conform to a cosine curve compared to LL conditions. This supports that a light cycle with a square wave caused some noise and made it difficult to detect periodicity. The similarity of expression pattern of clock gene-like contigs between lettuce and *Arabidopsis* seem to improve the reliability of determination of the subsistent genes because they had similar homology of alignment as well as expression pattern. In addition, the results suggest that detection of periodic contigs under both LL and LD conditions could reliably determine clock comprising genes by their periodic expression.

The functional categorization showed that the contigs related to the function of response to abiotic and biotic stimulus, response to stress and photosynthesis were the main part of the 215 common oscillating contigs (**Figure 4**). These results were particularly clarified by functional enrichment analysis (**Figure 5**). It is unsurprising that contigs related to the function of response to light stimulus were enriched because light stimulus is one effective external factor for circadian clocks (Barak et al., 2000; Alabadí et al., 2001; Fukuda et al., 2013). It is likely that response to blue light was significantly enriched. One likely reason is that *PHOT*s tended to express clear periodicity compared to *PHY*s and *CRY*s. The present study showed that 215 common oscillating contigs included both *PHOT1* and *PHOT2*. Actually, a previous study demonstrated that lettuce plants could receive low-intensity blue light and their circadian clock entrained to the weak blue light stimuli (Higashi et al., 2014) and both *PHOT1* and *PHOT2* also had clear periodic expression in tomato (Higashi et al., 2016). In contrast, *PHY*s and *CRY*s were not found to oscillate in either lettuce or tomato. Another reason is that some genes related to photosynthesis were also assigned the GO term for response to blue light. In rice, it was reported that stomatal opening, chloroplast movement and photosynthesis were activated by blue light signal (Lakshmanan et al., 2015), and these are major functions orchestrated by the circadian clock (Barak et al., 2000). Furthermore, recent studies indicated that the circadian clock regulates stress response (Harmer, 2009; Goodspeed et al., 2012; Lai et al., 2012; Marcolino-Gomes et al., 2014; Grundy et al., 2015). In the present study, oscillating contigs in lettuce showed similar results in that contigs related to the function of response to stress were significantly enriched. The enriched functions of the 215 common oscillating contigs such as response to light stimulus, response to stress and photosynthesis are important factors for plant production. This suggests that the 215 common oscillating contigs can be used as molecular markers for lettuce production. The valuable capabilities of molecular timetable methods can detect rhythm disorders and estimate the phase of the circadian clock from the expression profile of collective genes (Ueda et al., 2004). Therefore, it is possible to evaluate whether the gene expression pattern related to photosynthesis and optical response performs normally by analyzing periodicity of the 215 common oscillating contigs in lettuce. Moreover, a previous study showed that rhythm disorders and mismatching the phase of the circadian clock with the phase of environmental cycles caused poor growth in *Arabidopsis* (Dodd et al., 2005). Hence, the 215 common oscillating contigs may become molecular markers for determining optimal environmental cycles such as the LD ratio and temperature control in closed-type plant factories. This original approach has potential to be adapted to other cultivated plant with few genome resources and for application in closed-type plant factories.

We detected 976 contigs as LL-specific oscillating contigs and 144 as LD-specific oscillating contigs. There were 279 contigs detected as LL–LD common oscillating contigs, which were generated by circadian clocks in lettuce and BLAST results conclusively showed that 215 common oscillating contigs were targeted. These 215 oscillating contigs included clock gene-like contigs such as *CCA1* and *TOC1*, which showed similar expression patterns to those of *Arabidopsis* under both LL and LD conditions, and are assumed to have important roles in the circadian clock system. The GO Slim and GO Fat analyses showed that the GO terms of response to light stimulus, response to stress, photosynthesis and circadian rhythms were enriched in the 215 oscillating contigs and these terms were actually regulated by circadian clocks in several plants.

## Author Contributions

HF and TH designed this research. KA revised this article critically. AN and MH prepared a RNA-Seq library. TH performed data analysis. TH and HF wrote the manuscript. All authors discussed the results and implications and commented on the manuscript.

## Conflict of Interest Statement

The authors declare that the research was conducted in the absence of any commercial or financial relationships that could be construed as a potential conflict of interest.

## Footnotes

1 http://trace.ddbj.nig.ac.jp/DRASearch

2 http://www.ncbi.nlm.nih.gov/

3 http://diurnal.mocklerlab.org/diurnal_data_finders/new

4 https://www.arabidopsis.org

5 http://www.genome.jp/kegg/

6 https://david.ncifcrf.gov/home.jsp

7 http://www.cytoscape.org
